# Effect of Sinus Floor Augmentation with Platelet-Rich Fibrin Versus Allogeneic Bone Graft on Stability of One-Stage Dental Implants: A Split-Mouth Randomized Clinical Trial

**DOI:** 10.3390/ijerph19159569

**Published:** 2022-08-04

**Authors:** Aida Karagah, Reza Tabrizi, Parinaz Mohammadhosseinzade, Monirsadat Mirzadeh, Maryam Tofangchiha, Carlo Lajolo, Romeo Patini

**Affiliations:** 1 Department of Oral and Maxillofacial Surgery, School of Dentistry, Qazvin University of Medical Sciences, Qazvin 34199-15315, Iran; 2 Department of Oral and Maxillofacial Surgery, Shahid Beheshti University of Medical Sciences, Tehran 11151-19857, Iran; 3 Student Research Committee, Qazvin University of Medical Sciences, Qazvin 34199-15315, Iran; 4 Metabolic Diseases Research Center, Research Institute for Prevention of Non-Communicable Diseases, Qazvin University of Medical Sciences, Qazvin 34199-15315, Iran; 5 Department of Oral and Maxillofacial Radiology, Dental Caries Prevention Research Center, Qazvin University of Medical Sciences, Qazvin 34199-15315, Iran; 6 Department of Head, Neck and Sense Organs “Fondazione Policlinico Universitario A. Gemelli-IRCCS”, School of Dentistry, Catholic University of Sacred Heart, 00168 Rome, Italy

**Keywords:** sinus floor augmentation, platelet rich fibrin, allograft, freeze-dried bone allograft, implant stability

## Abstract

Rehabilitation of an edentulous posterior maxilla with dental implants is challenging, and sinus floor augmentation could be considered as an important surgical procedure for bone augmentation in this region before implant placement. Platelet-rich fibrin (PRF) is a new-generation platelet concentrate with simplified processing: its application in sinus floor augmentation has been widely investigated in literature. However, the biological properties and actual efficacy of this product remain controversial. This study assessed the effect of sinus floor augmentation with PRF versus freeze-dried bone allograft (FDBA) on stability of one-stage dental implants. This split-mouth randomized clinical trial evaluated 10 patients who required bilateral sinus floor augmentation. PRF and L-PRF membrane were used in one quadrant while FDBA and collagen membrane were used in the other quadrant. Implant stability was assessed by resonance frequency analysis (RFA) immediately, and 2, 4, and 6 months after implant placement. The implant stability quotient (ISQ) was compared over time and between the two groups using repeated measures ANOVA and independent sample *t*-test. The mean ISQ significantly increased over time in both groups (*p* < 0.001). The increase was greater in the PRF group (*p* < 0.05). Within the limitations of this study, PRF yielded superior results compared with FDBA regarding the stability of one-stage dental implants.

## 1. Introduction

Dental implants are a standard treatment option for replacement of lost teeth. Stability without micromovement is imperative for osseointegration of dental implants [1]. Generally, a 6- to 9-month healing period is required for implant placement if simultaneous with sinus floor augmentation [2]; however, surface treatments of new dental implants have shortened this period to approximately 6 months [3]. Implant stability depends on many factors, among which quality of bone plays a fundamental role. The mechanical properties, degree of mineralization, and remodeling capacity of bone determine the bone quality [4].

Inadequate bone height and insufficient width of the alveolar ridge may complicate implant placement in an atrophic maxilla. Ridge resorption and pneumatization of the maxillary sinus, as well as the poor quality of bone, may also make the implant rehabilitation of the posterior maxilla more challenging [5].

Sinus floor augmentation is a predictable and effective treatment procedure [6]. Several techniques have been suggested for vertical augmentation of the maxillary sinus, among which the classic lateral osteotomy is the most common, which was introduced by Tatum in 1976. The lateral augmentation procedure can be performed in one or two steps. The one-stage procedure refers to placement of dental implants simultaneous with sinus floor augmentation, while the two-stage procedure refers to placement of dental implants after bone healing [7]. Some differences exist in the graft materials used for bone augmentation and time of implant placement (simultaneous or delayed) in lateral sinus floor augmentation procedure [8]. In patients with severe atrophy of the maxillary alveolar ridge, sinus floor augmentation and implant placement are often performed in two separate phases. The healing time is at least 6 months when autogenous bone is used for sinus floor augmentation. In some cases, freeze-dried bone allograft (FDBA) is used to prevent donor site morbidity, which prolongs the healing period to approximately 8 months. Reduction of healing time is highly important to accelerate implant loading [9].

Platelet growth factors can enhance cell proliferation and healing of injured tissues [10]. Platelet-rich plasma (PRP) is a new tissue-engineered product. The autologous growth factors include transforming growth factor beta, platelet-derived growth factor (PDGF), insulin-like growth factor, and epidermal growth factor. Transforming growth factor beta and PDGF-AB can enhance soft tissue and bone healing by increasing the production of collagen and formation of primary callus [11]. Platelet-rich fibrin (PRF) is a second-generation platelet-derived product. It is a fibrin mesh containing leukocytes and cytokines and has angiogenesis and growth factor release potential similar to PDGF. It has been reported that PRF causes gradual release of autologous factors and is more effective than PRP in proliferation and differentiation of osteoblasts [12].

Studies on the efficacy of PRF for sinus floor augmentation are limited, and PRF and FDBA have not been previously compared for sinus floor augmentation. Thus, this study aimed to assess whether PRF can increase implant stability in implant placement simultaneous with sinus floor augmentation. For this purpose, the stability of dental implants placed simultaneously with sinus floor augmentation was compared in two groups using PRF and FDBA.

## 2. Materials and Methods

### 2.1. Trial Design

This split-mouth randomized clinical trial was conducted at the Oral and Maxillofacial Surgery Department of School of Dentistry, Qazvin University of Medical Sciences, from October 2018 to March 2020. The study was approved by the ethics committee of Qazvin University of Medical Sciences (IR.QUMS.REC.1399.327) and registered in the Iranian Registry of Clinical Trials (IRCT20191204045602N2).

### 2.2. Participants

The patients were selected among those presenting to the Oral and Maxillofacial Surgery Department of School of Dentistry, Qazvin University of Medical Sciences during the aforementioned period. A total of 10 partially edentulous patients who required bilateral maxillary sinus floor augmentation with a minimum of 6 months passed since the extraction of their first molars were enrolled after signing informed consent forms.

### 2.3. Inclusion Criteria

Table 1 presents the inclusion criteria for patients.

### 2.4. Exclusion Criteria

The exclusion criteria were systemic diseases that contraindicated implant placement such as uncontrolled diabetes mellitus, blood platelet disorders, serious osseous disorders, and cardiac arrhythmia; history of bone grafting in the posterior maxilla, immunocompromised patients, taking corticosteroids, taking aspirin before the procedure, positive history of chemotherapy and radiotherapy, and maxillary sinus pathologies.

### 2.5. Intervention

#### 2.5.1. Preparation of PRF

Before the surgical procedure, blood sample was collected from patients (2 syringes, 20 mL each) and transferred into intra-spin centrifuge tubes of Intra-Lock machine. The tubes were immediately centrifuged (Intra-Lock International Inc., Boca Raton, FL, USA) at 2800 rpm for 12 min. Next, the tubes were placed in a sterile tube holder, and the fibrin matrix was obtained by separating the blood clot and red blood cells from the leukocyte-PRF (L-PRF). The L-PRF was placed in Xpression tray for 5 min to prepare the PRF membrane [14].

#### 2.5.2. Surgical Procedure

All patients received 2 g amoxicillin as a prophylaxis 1 h before the procedure. Those allergic to amoxicillin received 600 mg clindamycin instead. After the induction of local anesthesia, a crestal incision was made with an anterior releasing incision in both sides of the maxilla. A full-thickness mucoperiosteal flap was elevated. Sinus floor augmentation was performed bilaterally for each patient by the lateral approach using Tola Sinus Kit (TOLA, Surgident, Korea) to create a lateral window safely, preferably without perforating the Schneiderian membrane. The Schneiderian membrane was then lifted bilaterally.

To reinforce the Schneiderian membrane, a PRF membrane measuring 20 × 20 mm was used at one side, and a collagen membrane (CenoMembrane; Hamanand Saz Baft Kish Co., Tehran, Iran) measuring 20 × 20 mm with 0.2 to 0.6 mm thickness was used at the other side. L-PRF was used on the PRF side while FDBA with particles measuring 500–1000 µm in size (Cenobone cortical cancellous powder; Hamanand Saz Baft Kish Co., Tehran, Iran) was applied on the other side. In each patient, a similar volume of materials was used on both sides.

Dental implants with 4.5 mm diameter and 10 mm length (BEGO implant system GmbH & Co. KG, Bremen, Germany) were then placed with the standard protocol according to the manufacturer’s guidelines (800 rpm, 20 N/cm torque). The healing abutments were then tightened at both sides. Immediately after placing the graft material, a resorbable collagen membrane (CenoMembrane; Hamanand Saz Baft Kish Co., Tehran, Iran) was used over the FDBA site and a L-PRF membrane was used over the PRF site to cover the sinus window. Finally, the area was sutured with 3/0 surgical absorbable suture (braided poly-glycolic acid, reverse cut, 3/8 circle; Pezeshkyaran Amin, Iran). After surgery, the patients were requested to apply a cold compress over the surgical site, and were prescribed 675 mg co-amoxiclav (n = 20) every 8 h for 1 week, 400 mg ibuprofen every 6 h for 4 days, and 0.12% chlorhexidine mouthwash for one week. The sutures were removed after 7 days. Panoramic radiographs were taken immediately after surgery and at 6 months postoperatively.

#### 2.5.3. Outcomes

Assessment of stability: two examiners, blinded about the treatment process and group allocation of the quadrants, were trained to measure the stability of dental implants. Stability was measured by the resonance frequency analysis (RFA) using Osstell (Osstell, Gothenburg, Sweden). For this purpose, the transducer (smart peg/type 26) was connected to the fixture in mesiodistal and buccolingual directions and the RFA was conducted; the mean value was recorded as the implant stability quotient (ISQ) [14]. The ISQ was measured immediately after implant placement and 2, 4, and 6 months later. The data were compared between the two sides.

### 2.6. Sample Size Calculation

The sample size was calculated to be 10 sites in each group according to a study by Tabrizi et al. [14], assuming alpha = 0.05, study power of 80%, standard deviation of ISQ to be 3.64 and 3.42 in the two groups, and considering a 20% loss to follow-up.

### 2.7. Randomization

Randomization was performed by using sealed envelopes and a Random Allocation Software program (version 1.0.0, Isfahan, Iran). Accordingly, the treatment sequence and the quadrant receiving PRF were randomly selected in each patient by a dental assistant not involved in the study.

### 2.8. Blinding

Both examiners and the statistician who analyzed the data were blinded about the group allocation of the quadrants.

### 2.9. Statistical Analysis

Data were analyzed using SPSS version 23 (SPSS Inc., Chicago, IL, USA). The mean and standard deviation values were reported for the variables, and stability was compared between the two groups and at different time points using independent sample *t*-test and repeated measures ANOVA at *p* < 0.05 level of significance. An inter-examiner reliability analysis was performed using the kappa statistic to determine the level of agreement between the two examiners.

## 3. Results

Ten patients, including 7 females and 3 males, requiring bilateral augmentation of the maxillary sinus were enrolled. The mean age of patients was 48.3 ± 8.31 years. Bilateral sinus floor augmentation was performed for each patient using PRF on one side and FDBA on the other side. Implant stability was measured by two examiners immediately after surgery, and after 2, 4, and 6 months. Ten implants were placed in each of the PRF and FDBA groups.

There were two cases of membrane perforation in this study. In one of them, L-PRF membrane was used to reinforce the Schneiderian membrane, and in the other, collagen membrane was used; the results of vertical augmentation of bone in these two cases were similar to the results in cases without perforation. PRF was used to repair the sinus perforation in one side since it has self-adhesion properties and does not require suturing.

**Figure 1:**
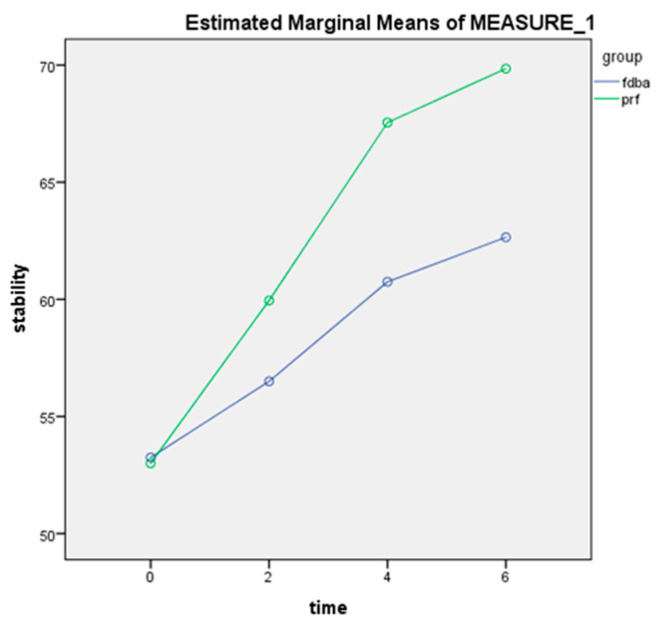
Trend of change in the mean ISQ over time in the two groups. As shown, the mean ISQ increased over time in both groups; however, this increase was greater in the PRF group.

Table 2 shows the mean ISQ values in the two groups at different time points. According to independent *t*-test, the mean ISQ measured immediately after implant placement was not significantly different between the two groups (*p* = 0.73). However, the mean ISQ was significantly higher in the PRF group at 2 months (*p* = 0.002), 4 months (*p* = 0.001), and 6 months (*p* = 0.001). As shown in Figure 1, repeated measures ANOVA indicated a significant change in ISQ over time in both groups (*p* < 0.001). As shown, the ISQ was higher in the PRF group than the FDBA group as measured by both examiners (*p* < 0.001). The LSD post-hoc test revealed significant differences in all pairwise comparisons of the time points in both groups such that the ISQ significantly increased over time (at each time point compared with the previous time point) in both groups (*p* < 0.001). According to the amount of partial eta squared, 94% of the accuracy of the stability model was due to the intervention. The inter-examiner reliability was calculated to be kappa = 0.99 (0.985–0.993), indicating excellent agreement between the two examiners.

## 4. Discussion

Sinus floor augmentation is a commonly practiced surgical procedure with a relatively long healing time due to the low vascularization rate of the sinus cavity. For this reason, PRF is increasingly used to promote vascularization and enhance wound healing [15]. This study assessed the effect of sinus floor augmentation with PRF versus FDBA on stability of one-stage dental implants. The results showed that the mean ISQ significantly increased over time in both groups (*p* < 0.001). However, this increase was significantly greater in the PRF group (*p* < 0.05).

Maxillary sinus floor augmentation by the lateral approach is a commonly practiced technique with a high implant survival rate [16]. However, damage to the Schneiderian membrane is the most common side effect of such a procedure. Post-surgical complications include surgical site infection, abscess, dehiscence, purulent discharge, maxillary sinusitis, graft exposure, and graft loss [17].

Different graft materials have been used over the years to obtain more favorable results. Bone substitutes such as xenografts and allografts are more commonly used than autografts for sinus floor augmentation, mainly due to the fact that they cause fewer postoperative complications since donor site morbidity is the main complication of autografts. Several studies have reported successful use of xenografts and allografts. Nonetheless, they increase the cost of treatment. Additionally, risk of disease transmission is still a concern [18]. In recent years, PRF, alone or in combination with different materials, has been proposed as an alternative material for sinus floor augmentation.

Measurement of ISQ by RFA provides beneficial clinical information regarding the implant–bone connection over the course of a treatment or during the follow-up period. Additionally, RFA is used to assess stability as a function of implant–bone interface, but it is affected by a number of factors such as the healing time, bone density, and the length of exposed part of the implant above the alveolar crest. Moreover, implant osseointegration is affected by the surface topography and physicochemical properties of dental implants [14]. Additionally, growth factors play a role in the improvement of bone–implant contact since the osteoinductive effects of bone morphogenetic proteins and TGF-β on peri-implant bone healing have been well documented [19,20]. Therefore, PRF, in combination with other products, was used in some studies. For example, Choukroun et al. [21] used PRF in combination with FDBA. Histological analysis indicated a sufficient amount of newly formed mineralized trabecular bone, which was rich in osteocytes with prominent osteoid borders in contact with dense cellular osteoblasts in the front. This combination also decreased the wound-healing period [21]. However, another study showed that the addition of PRP to the conventional bone substitutes in sinus grafting had no histologically detectable advantage with regard to bone healing or remodeling [22]. Tanaka et al. [23] reported that the addition of PRF to deproteinized bovine bone histologically increased new bone formation. Nonetheless, they also reported that the addition of granular materials could increase the risk of infection. Accordingly, sinus floor augmentation with the use of PRF alone was recently introduced.

A systematic review by Sherif Ali et al. [24] reported that three studies had used PRF alone for augmentation. They reviewed a total of 57 sinus floor augmentation procedures and placement of 110 implants in 46 patients. The preoperative radiographic residual bone height was 1.5 to 6.1 mm. Sinus floor augmentation had been performed with the lateral approach, and dental implants had been placed simultaneously in all reviewed articles. Moreover, all of them reported that the authors place the PRF clot within the sinus cavity [24]. Mazor et al. [25] and Simon Peri et al. [26] placed one or two PRF membranes over the Schneiderian membrane and the lateral window, while Tajima et al. [27] did not use any membrane [25,26,27]. Implant loading was scheduled 6 months after the first surgical procedure, and no complications occurred during the healing period. Perforation occurred in 5.3% of the cases (3 patients), which was easily managed by the PRF membrane placement. At the time of abutment placement, 110 implants were completely stable [24]. Among the aforementioned studies, Tajima et al. [27] used Osstell to perform RFA and reported the mean ISQ to be 66.5 ± 6.15 at the time of second-stage surgery (after 6 months). Moreover, the radiographic assessment indicated a mean bone gain of 9.8 mm [27]. Histological and histomorphometric assessments were made by Mazor et al. [25] that obtained bone biopsy samples during the second-stage surgery. Histopathological analyses indicated viable, completely organized bone with a trabecular pattern and dense collagen matrix. Additionally, osteoblasts and osteocytes were easily detectable in the lacunae. Histomorphometric analyses indicated over 39% bone matrix with a mean value of 33 ± 5%. In two-stage sinus floor augmentation surgery with delayed implant placement, it is the graft materials’ task to preserve the position of the Schneiderian membrane. It has been reported that PRF is gradually resorbed. Thus, PRF alone may not be sufficient to preserve the membrane without simultaneous implant placement [27]. However, Aoki et al. [28] reported different evidence and indicated that PRF clots alone had the capacity to create a space between the original sinus floor and the elevated Schneiderian membrane to allow new bone formation. The volume of PRF may also be important to preserve the membrane [29]. According to a systematic review conducted in 2020, no comparisons have been made so far between the use of platelet concentrates as grafting material and the routinely used biomaterials, such as allografts and xenografts, in sinus floor augmentation [29]. This statement highlights the novelty of using PRF for this purpose.

The present study compared the effects of PRF and FDBA, along with collagen membrane on the stability of implants placed simultaneously with sinus floor augmentation in a one-stage procedure. The enrolled patients had no systemic disease since systemic conditions can affect bone formation or cause bone resorption. This was in line with an 11-year retrospective study conducted by Moy et al. [30], which suggested systemic diseases as an important risk factor for implant failure [30]. Moreover, patients with parafunctional habits such as bruxism and clenching were excluded from the study and oral hygiene of patients was evaluated pre- and postoperatively and during the follow-up sessions, which was in agreement with the study by Porter and von Fraunhofer [31]. Patients with maxillary sinus pathologies were also excluded, in accordance with the study by Torretta et al. [32]. According to Trieger et al. [33], Laskin et al. [34], and Sharaf et al. [35], antibiotic therapy decreases the risk of implant failure. Thus, antibiotics were prescribed pre- and postoperatively in the present study [33,35].

The lateral window technique was adopted in this study to elevate the sinus membrane, which showed 100% success rate with no serious side effect at 6 months; this finding was in agreement with the results of Wallace et al. [16]. Tolla II sinus lift surgical kit was used in this study to increase the level of safety and predictability of the results. This technique decreases the risk of perforation due to the presence of a stopper and minimizes patient discomfort and the complications related to the sinus floor augmentation procedure. In the present study, of 20 sinus floor augmentation procedures, sinus perforation occurred in 2; out of which, 1 belonged to the L-PRF and the other to the FDBA and collagen membrane group. The Schneiderian membrane was successfully reinforced with L-PRF membrane in PRF group and with collagen membrane in FDBA group, with no side effect. Clinical examinations indicated optimal primary stability of all implants in both groups. Implant stability increased with time, and such an increase was greater in the PRF group. Choukroun et al. [21] evaluated the efficacy of PRF and FDBA for sinus floor augmentation. They reported histologically similar results in PRF + FDBA group at 4 months and in FDBA monotherapy group at 8 months. They concluded that the combination of FDBA and PRF would decrease the waiting time after sinus floor augmentation procedure [21].

In the present study, the mean ISQ at 4 months in the PRF group was over 67% (67.55%) according to both observers, while the mean value was 60.75% in the FDBA group at 4 months (Table 3). This result was in line with that of Choukroun et al. [21]. In the PRF group, the mean ISQ at 6 months was 69.85% according to both observers, while this value reached 62.65% at 6 months in the FDBA group.

In the present study, the mean ISQ measured by the two observers at 6 months was ≥70 in 15 out of 20 patients. In the FDBA group, no case of stability > 70 was noted. The remaining five cases in the PRF and 5 cases in the FDBA group had an ISQ between 65–69. Among the remaining 15 cases in the FDBA group, 14 had an ISQ between 60–64 and one case had an ISQ < 60 (Table 4). In the FDBA group, a greater amount of time was required to obtain adequate stability for loading. This conclusion was in agreement with the results of Chirila et al. [36] and Uncu and Alaaddinoglu [1], who stated that the required time until implant loading was 6 to 9 months [1,36]. Sennerby et al. [37] reported that measurement of ISQ could serve as a suitable parameter for assessment of implant stability and decision making during the course of treatment and the follow-up period. Measurement of stability is influenced by the topographic and physiochemical properties of dental implants. They used a classification system with three colored zones: green, yellow, and red. The green zone indicated implant stability of 70 and higher, which was considered as high stability. Implants with high stability are considered safe for immediate loading. The red zone indicated questionable implants with ISQ < 55, which indicated low stability of implants. The yellow zone indicated ISQ between 55–70, indicating moderate stability [37]. Michael et al. [38] suggested that implants with an ISQ < 65 require more time for loading and should be rechecked after 3 weeks; loading can be performed when the ISQ value reaches >65 [38].

One drawback of PRF is that it requires preoperative clinical procedures. However, it can be easily prepared. The clinician only needs to draw some venous blood and centrifuge it in the respective device. An advantage of PRF against whole blood is that it decreases the risk of perforation of the sinus membrane during the preparation of the site that will host the dental implant; in fact, the PRF, acting as a stabilizer of the already elevated sinus membrane, has an intrinsic stiffness that acts as a bearing protector and separator between the sinus membrane and the surgical drill [39].

Considering the lower cost, the easier preparation, the autogenic nature of PRF, and its optimal efficacy in decreasing the waiting time for loading, it can be used as an alternative bone substitute for sinus floor augmentation and simultaneous implant placement.

Limitations: The main limitation of this study was the difficulty to select patients with similar anatomical properties in their right and left quadrants. Accordingly, care was taken to select patients with similar alveolar bone height and width in their right and left maxillary quadrants as much as possible.

Large-scale studies are still required to validate these preliminary results. Further clinical studies with larger sample size are required to confirm the reduction of waiting time for implant loading by using PRF. Studies with longer follow-ups and post-loading assessments are also required to obtain more reliable clinical results.

## 5. Conclusions

Within the limitations of this study, the results showed the superiority of PRF to FDBA regarding the stability over time of one-stage dental implants placed after maxillary sinus lifting. Regarding implant stability, PRF showed promising results as a sole filling material in sinus floor augmentation with simultaneous implant placement.

## Figures and Tables

**Table 1:** Inclusion criteria of patients.

Criterion	Range and Unit of Measurement
Patients requiring bilateral maxillary sinus floor augmentation based on cone-beam computed tomography scans	-
Inadequate residual bone height	A minimum of 4 mm and maximum of 8 mm of residual bone height between the alveolar crest and the sinus floor
Adequate inter-occlusal space that could accommodate the implant abutment and the future restoration	Inter-maxillary (occlusal) space: >10 mm
Adequate mesiodistal space that could accommodate the implant abutment and the future restoration	Mesiodistal space (interdental space): ≥7 mm (for 1 implant)
Adequate buccopalatal space	Buccopalatal space ≥6 mm
Willingness to receive dental implant and consenting to the related procedures	-
Oral hygiene according to Silness and Loe plaque index [13]	Those with a plaque index score of 0 (absence of plaque) and 1 (low amount of plaque) were enrolled.

**Table 2:** Mean ISQ in the two groups at baseline and at 2, 4, and 4 months after treatment in the two.

Time	Group 1 FDBA		Group 2 PRF		CI 95%	*p* Value
	Mean	SD	Mean	SD		
Baseline	53.25	2.268	53.00	2.384	−1.2:1.7	0.73
2 months	56.50	3.171	59.95	3.284	−5.5:−1.3	0.002
4 months	60.75	2.573	67.55	1.791	−8.2:−5.3	0.001
6 months	62.65	2.110	69.85	2.059	−8.5:−5.8	0.001

**Table 3:** Interpretation of data according to the Osstell’s clinical guidelines at 4 months.

	≥70	69–65	64–60	<60
PRF	15%	65%	2%	0
FDBA	0	0	65%	35%

**Table 4:** Interpretation of data according to the Osstell’s clinical guidelines at 6 months.

Group	≥70	69–65	64–60	<60
PRF	75%	25%	0	0
FDBA	0	25%	70%	5%

## Data Availability

Not applicable.
